# Dietary Macronutrient Composition Differentially Modulates the Remodeling of Mitochondrial Oxidative Metabolism during NAFLD

**DOI:** 10.3390/metabo11050272

**Published:** 2021-04-26

**Authors:** Nathan Kattapuram, Christine Zhang, Muhammed S. Muyyarikkandy, Chaitra Surugihalli, Vaishna Muralidaran, Tabitha Gregory, Nishanth E. Sunny

**Affiliations:** Department of Animal and Avian Sciences, University of Maryland, College Park, MD 20742, USA; nmkatta@umd.edu (N.K.); cyzhang@terpmail.umd.edu (C.Z.); mshafeekhmk@gmail.com (M.S.M.); csurugi@umd.edu (C.S.); vmurali1@umd.edu (V.M.); tgregory@terpmail.umd.edu (T.G.)

**Keywords:** mitochondria, fatty liver, lipogenesis, targeted metabolomics, mass spectrometry, lipid oxidation

## Abstract

Diets rich in fats and carbohydrates aggravate non-alcoholic fatty liver disease (NAFLD), of which mitochondrial dysfunction is a central feature. It is not clear whether a high-carbohydrate driven ‘lipogenic’ diet differentially affects mitochondrial oxidative remodeling compared to a high-fat driven ‘oxidative’ environment. We hypothesized that the high-fat driven ‘oxidative’ environment will chronically sustain mitochondrial oxidative function, hastening metabolic dysfunction during NAFLD. Mice (C57BL/6NJ) were reared on a low-fat (LF; 10% fat calories), high-fat (HF; 60% fat calories), or high-fructose/high-fat (HFr/HF; 25% fat and 34.9% fructose calories) diet for 10 weeks. De novo lipogenesis was determined by measuring the incorporation of deuterium from D_2_O into newly synthesized liver lipids using nuclear magnetic resonance (NMR) spectroscopy. Hepatic mitochondrial metabolism was profiled under fed and fasted states by the incubation of isolated mitochondria with [^13^C_3_]pyruvate, targeted metabolomics of tricarboxylic acid (TCA) cycle intermediates, estimates of oxidative phosphorylation (OXPHOS), and hepatic gene and protein expression. De novo lipogenesis was higher in the HFr/HF mice compared to their HF counterparts. Contrary to our expectations, hepatic oxidative function after fasting was induced in the HFr/HF group. This differential induction of mitochondrial oxidative function by the high fructose-driven ‘lipogenic’ environment could influence the progressive severity of hepatic insulin resistance.

## 1. Introduction

Non-alcoholic fatty liver disease (NAFLD) is a common co-morbidity of obesity and is prevalent in over 70% of patients with type-2 diabetes mellitus (T2DM) [1,2]. Lipid accumulation in the liver is a characteristic feature of NAFLD, due to alterations in carbohydrate and lipid metabolism [3,4,5,6]. Mitochondria are highly dynamic cellular organelles, which integrate macronutrient oxidation, cellular respiration, and ATP synthesis with anabolic networks including gluconeogenesis and de novo lipogenesis. The cellular oxidative processes can account for as much as 90% of whole-body oxygen consumption [7,8,9] with the mitochondria accounting for the vast majority. With the onset of insulin resistance and NAFLD, mitochondrial oxidative networks (e.g., ketogenesis, tricarboxylic acid (TCA) cycle, β-oxidation, oxidative phosphorylation (OXPHOS), and ATP synthesis) progressively become dysfunctional, contributing to the accumulation of lipids in the liver and ensuing ‘lipotoxicity’ [3,4,5,10,11]. Thus, mitochondrial oxidative dysfunction is a central feature of insulin resistance and NAFLD.

Under normal physiology, the metabolic plasticity of the liver allows it to efficiently switch from lipid storage mechanisms, during fed conditions, to lipid oxidation during fasting. Approximately 60% of the triglycerides in a human liver arise from the adipose tissue derived plasma non-esterified fatty acid (NEFA) pool, with new lipid synthesis from carbohydrates (de novo lipogenesis) and free fatty acids from the diets contributing around 25% and 15% respectively [12]. While de novo lipogenesis has a relatively minor contribution to lipid accumulation in the liver under normal physiology, its contribution increases significantly during NAFLD [13,14,15]. Together with the dysfunctional remodeling of the hepatic mitochondrial oxidative networks, higher rates of de novo lipogenesis contribute to the progressive severity of insulin resistance and NAFLD [13,16,17].

Chronic exposure to diets rich in fat/lipid calories or refined carbohydrate calories (usually from fructose) are common strategies to induce insulin resistance and NAFLD in rodent models [4,17,18]. These extreme macronutrient exposures to either a high-fat or a high-fructose environment are bound to differentially impact the activity of multiple hepatic metabolic networks. For example, high dietary fatty acids can suppress de novo lipogenesis in the liver [19,20], while a high-fructose diet induces de novo lipogenesis [21,22,23]. Furthermore, a lipid-rich environment is known to induce mitochondrial oxidative networks during the etiology of NAFLD [18,24,25]. A combination of high fructose and high lipids in the diet has also been shown to exacerbate NAFLD [26,27]. Interestingly enough, mitochondrial oxidative dysfunction is a common feature of both high-fat and high-fructose-induced NAFLD [18,21]. Most Western diets consist of high lipid calories, together with significant calories from refined carbohydrates [28]. The macronutrient composition of a Western diet is different from the extremes of both a high-fat and high-fructose calorie diet, which are commonly used to induce NAFLD in rodent models. Furthermore, it is unclear whether a dietary environment rich in both lipids and carbohydrates remodels mitochondrial oxidative function similarly to the remodeling observed with high-fat diet (60% fat calories) induced NAFLD [18,25]. A custom diet containing 25% fat calories and 34.9% fructose calories was developed to simulate a Western diet. Our objective was to induce hepatic de novo lipogenesis and mild NAFLD in the rodent liver by exposure to this high-fructose/high-fat diet for 10 weeks and, investigate whether this resulted in differential remodeling of mitochondrial oxidative metabolism, compared to the hepatic metabolic milieu resulting from high-fat diet exposure.

## 2. Results

### 2.1. Phenotypic Characteristics for the Mice Reared on Low-Fat (LF), High-Fat (HF), and High-Fructose/High-Fat (HFr/HF) Diets

Diets high in lipids (HF) or fructose calories are commonly used to induce insulin resistance and NAFLD in rodent models. Our HFr/HF diet was customized to contain higher calories from fructose along with 2.5 time more lipids than the LF diet. We rationalized that the HFr/HF macronutrient environment is more reflective of the Western diets consumed by humans relative to the HF diet. The metabolic characteristics in rodents following the consumption of these diets for 10 weeks are presented in Table 1. As expected, under both fed and fasting conditions, the mice on HF and HFr/HF diets had significantly higher body weights than their LF counterparts (*p* ≤ 0.05). Furthermore, the body weights of the HF mice were significantly higher than the HFr/HF mice (g; 42.9 ± 1.1 vs. 33.4 ± 1.1; *p* ≤ 0.05). Liver weights were higher in the HF (g; 1.84 ± 0.18; *p* ≤ 0.05) and HFr/HF groups (g; 1.74 ± 0.08; *p* ≤ 0.05) relative to the LF group (g; 1.43 ± 0.05) under fed conditions, with the weights remaining similar between HF and HFr/HF groups. No significant differences in liver weights were observed after overnight fast between any of the three groups. While fed plasma glucose remained similar between groups, fasted plasma glucose was higher in the mice reared on a HF diet compared to those reared on a LF diet (mM; 5.99 ± 0.33 vs. 4.76 ± 0.36; *p* ≤ 0.05). The HFr/HF group had the highest hepatic glycogen levels under fed conditions (mg/g liver; HFr/HF, 64.3 ± 3.4 vs. HF, 50.5 ± 4.5; *p* ≤ 0.05). There was a non-significant increase in fed plasma insulin following HF diet (ng/mL; 3.83 ± 1.13) and HFr/HF diet (ng/mL; 2.92 ± 1.20) feeding when compared to the LF diets (1.24 ± 0.40). Fasted plasma insulin remained similar between groups. Plasma non-esterified free fatty acids (NEFA) under fed conditions were significantly higher in the HF group (mM; 0.52 ± 0.03; *p* ≤ 0.05) compared to both LF (mM; 0.34 ± 0.05) and HFr/HF groups (mM; 0.37 ± 0.04). However, following an overnight fast, NEFA levels were slightly higher (non-significant) in the LF group (mM; 0.92 ± 0.04), compared to both HF (mM; 0.78 ± 0.04) and HFr/HF group (mM; 0.78 ± 0.05). The fed to fasted change in NEFA levels illustrate the blunted induction of lipolysis in both the HF and HFr/HF mice. Under fed conditions, liver triglycerides were higher in the high-fat fed mice (mg/g liver; 31.1 ± 2.7; *p* ≤ 0.05) compared to both LF (mg/g liver; 23.1 ± 2.0) and HFr/HF groups (mg/g liver; 23.5 ± 1.7). Following fasting, liver triglycerides remained similar between groups. Both HF and HFr/HF mice had bigger deposits of inguinal adipose tissue under fed and fasted conditions. The adipose tissue weight in the HF mice were significantly higher than the HFr/HF group under fed conditions (g; 2.74 ± 0.25 vs. 1.56 ± 0.15; *p* ≤ 0.05). Gene expression profiles of common markers of inflammation, profiled under fed conditions, (*Tnfa*, *Il6*, and *Nfkbia*; Figure S1) did not show significant difference between the three groups. Taken together with the metabolic characteristics in Table 1, these results are indicative of a mild onset of insulin resistance in both the HF and HFr/HF mice relative to their LF counterparts, reflecting early stages of NAFLD.

### 2.2. Hepatic De Novo Lipogenesis Was Higher in Mice Fed High-Fructose/High-Fat Diet Compared to those Fed a High-Fat Diet.

**Figure 1:**
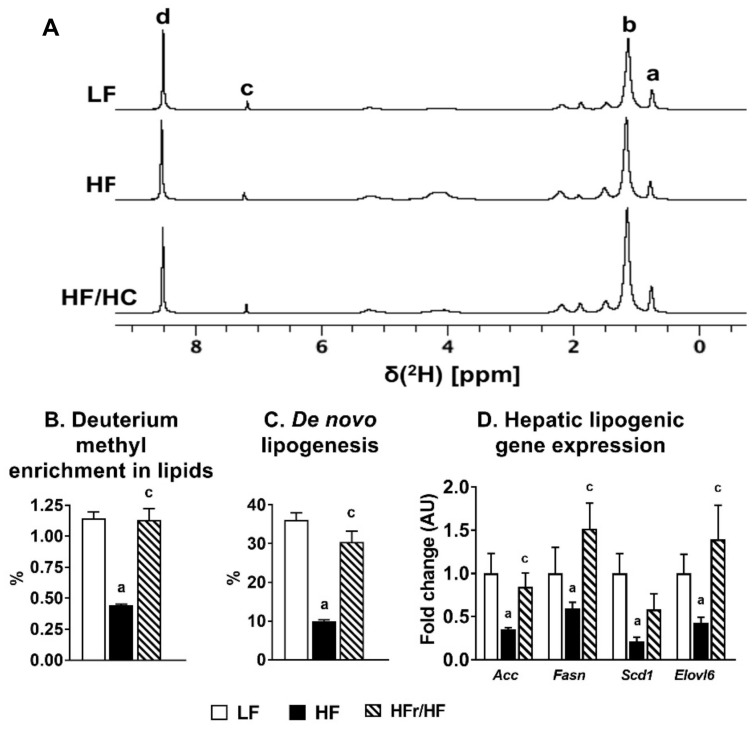
De novo lipogenesis was induced in the liver of mice fed the HFr/HF diet. (**A**) 2H NMR spectra of total lipids in the liver, assignments of peaks on the spectrum are (a) ω-3 methyl, (b) aliphatic chain, (c) chloroform, and (d) pyrazine standard. Deuterium enrichment of the methyl peaks provide the qualitative information for the calculation of de novo lipogenesis. (**B**) Deuterium enrichment of the methyl groups in lipids extracted from the liver, (**C**) De novo lipogenesis in the liver, and (**D**) Lipogenic gene expression profile in the liver. Results (*n* = 5/group) were considered significant at *p* ≤ 0.05 following pairwise mean comparisons, which are represented by the following alphabets. ‘a’—LF vs. HF; ‘c’—HF vs. HFr/HF.

We determined whether the two macronutrient compositions (HF vs. HFr/HF), which were both predicted to induce insulin resistance and NAFLD, differ in their ability to induce hepatic de novo lipogenesis. Based on the deuterium incorporation into total lipids, the representative snap shots of which are provided in Figure 1A, it is clear that the deuterium enrichment in the terminal methyl group of the lipids is lower in the HF groups compared to their LF counterparts (Figure 1B). Feeding on the HFr/HF diet results in an increase in this methyl group enrichment relative to the HF diet fed mice (Figure 1B.). These measurements translated into significantly lower rates of de novo lipogenesis in the HF fed mice compared to the LF group and significantly higher rates of de novo lipogenesis in the HFr/HF mice compared to the HF group (Figure 1C). A similar trend was evident from the expression patterns of key genes involved in lipid synthesis including *ACC*, *FASN*, *SCD1*, and *ELOVL6*, where their expression levels were higher in the liver of HFr/HF mice compared to their HF counterparts (Figure 1D). Overall, these results point to a clear induction of hepatic de novo lipogenesis following HFr/HF feeding, and furthermore, the strong suppression of hepatic de novo lipogenesis by the lipid-rich dietary environment (HF).

### 2.3. Differences in Diet Composition Alter AKT Phosphorylation and Hepatic Mitochondrial Protein Expression during Fed and Overnight Fasting Conditions

**Figure 2:**
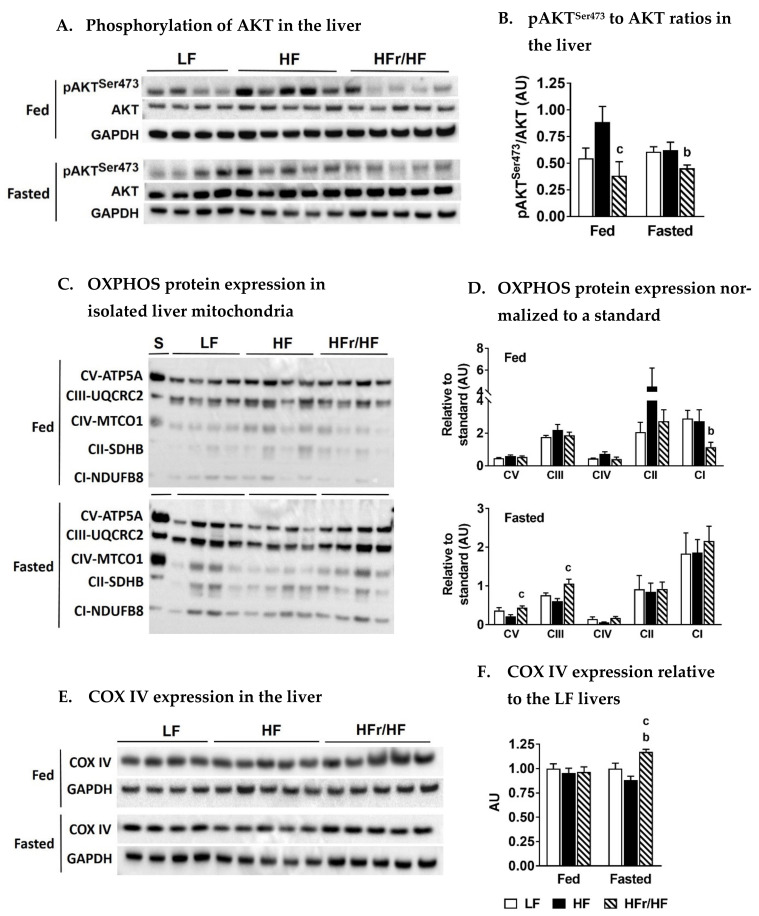
Phosphorylation of AKT and expression of mitochondrial proteins in the liver following HF and HFr/HF feeding. (**A**) Western blot analysis of AKT phosphorylation in the liver following feeding and overnight fasting, (**B**) Plot of pAKT^Ser473^ to AKT ratios in the liver, (**C**) Expression of OXPHOS proteins in isolated liver mitochondria from fed and fasted mice, (**D**) Densitometry plot of OXPHOS protein expression, normalized to a mitochondrial protein standard, (**E**) Expression of the mitochondrial protein COX IV in the liver tissue of fed and fasted mice, (**F**) Plot of COX IV expression in HF and HFr/HF livers normalized to their LF counterparts. Results (*n* = 4–5/group) were considered significant at *p* ≤ 0.05 following pairwise mean comparisons, which are represented by the following alphabets. ‘b’—LF vs. HFr/HF; ‘c’—HF vs. HFr/HF.

The phosphorylation of AKT was higher in the mice on an HF diet under fed conditions relative to the HFr/HF group (Figure 2A,B). Under fasting conditions, the phosphorylation rates of AKT remained similar between LF and HF groups, but they tended to be higher than those in HFr/HF livers (Figure 2A,B). Then, we determined whether the varying macronutrient environment differentially impacts mitochondrial protein expression.

Under fed conditions, the mitochondrial OXPHOS protein expression for Complex I subunit in the HFr/HF group was lower than both LF and HF groups (Figure 2C,D). On the contrary, following an overnight fast, Complex III and V were all higher expressed (*p* ≤ 0.05) in the HFr/HF mitochondria compared to the HF group (Figure 2C,D). This trend following an overnight fast was also evident for COX IV expression in the liver tissue where HFr/HF mice had higher levels (*p* ≤ 0.05) of COX IV compared to the HF mice (Figure 2E,F). Taken together, the changes in expression of mitochondrial proteins between the three groups could indicate alterations in hepatic mitochondrial content.

### 2.4. Switch from Carbohydrate to Free Fatty Acid Utilization during Feeding to Fasting Transition in HF and HFr/HF Mice

**Figure 3:**
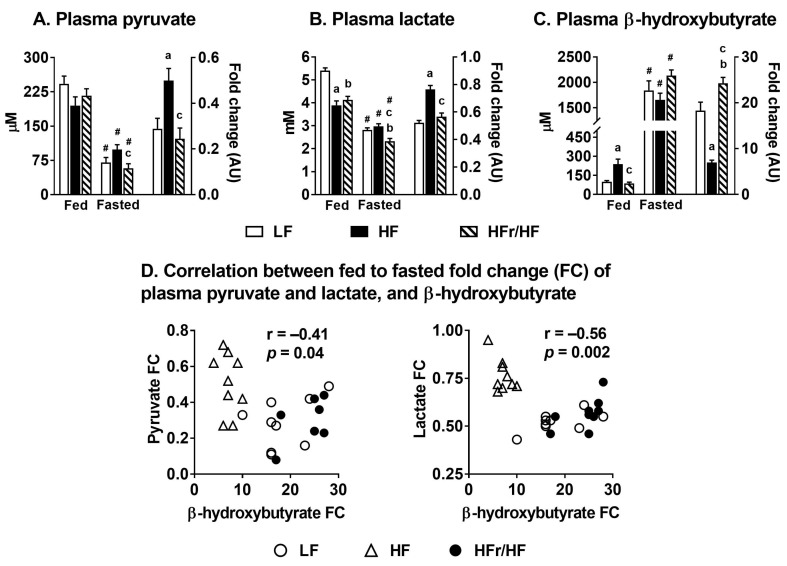
Fed to fasting switch from carbohydrate oxidation to free fatty acid oxidation following HF and HFr/HF feeding. Changes in plasma concentrations during fed and overnight fasted conditions in (**A**) Pyruvate, (**B**) Lactate, and (**C**) β-hydroxybutyrate. (**D**) Correlations between fed to fasted fold changes of plasma pyruvate and plasma lactate, and the fed to fasted fold changes of β-hydroxybutyrate. Results (*n* = 7–9/group) were considered significant at *p* ≤ 0.05 following pairwise mean comparisons, which are represented by the following letters. ‘a’—LF vs. HF; ‘b’—LF vs. HFr/HF; ‘c’—HF vs. HFr/HF. ‘#’ indicates significance at *p* ≤ 0.05 following a *t*-test between fed and fasted.

Levels of pyruvate, lactate, and β-hydroxybutyrate in plasma were determined using gas-chromatography mass-spectrometry (GC-MS) under fed and fasted conditions. This allowed us to determine whether HF vs. HFr/HF feeding altered the efficiency to switch from utilizing carbohydrates during the fed state to utilizing free fatty acids during fasted states. Fasting significantly decreased (*p* ≤ 0.05) both pyruvate and lactate levels in all the three dietary treatment groups (Figure 3A,B). However, the fold change in the fasting-mediated decrease in plasma lactate and pyruvate levels was significantly higher in the HFr/HF group compared to the HF group (Figure 3A,B).

Furthermore, fasting significantly induced plasma β-hydroxybutyrate levels in all three groups (Figure 3C). However, the fasting-mediated induction in β-hydroxybutyrate was significantly higher in the HFr/HF group compared to their HF counterparts (Figure 3C). Then, we tested the correlation between the fasting-induced fold changes in pyruvate and lactate with that of β-hydroxybutyrate. These correlation plots (Figure 3D) illustrate a significant negative correlation (*p* ≤ 0.05), suggesting that the mice reared on the HF diet were less efficient in switching from carbohydrate utilization during fed conditions to free fatty acid utilization during fasting conditions.

### 2.5. Profiles of Metabolic Intermediates in the Liver Tissue Point to Differences in Mitochondrial Metabolism between Mice Reared on HF and HFr/HF Diets

**Figure 4:**
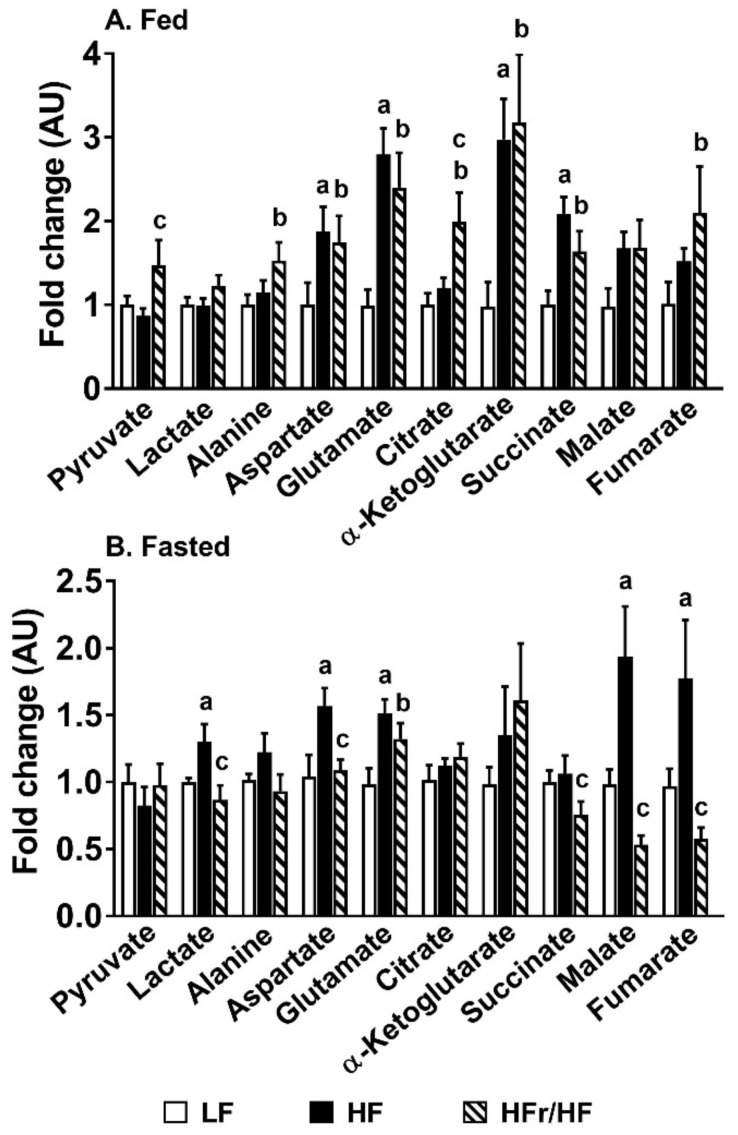
Profiles of organic acids and amino acids in the liver of mice reared on HF and HFr/HF diets. (**A**) Fed and (**B**) overnight fasted profiles of organic acids and amino acids in the liver. Data are presented as fold changes in metabolites in the liver of HF and HFr/HF livers relative to their age-matched LF counterparts. Results (*n* = 7–11/group) were considered significant at *p* ≤ 0.05 following pairwise mean comparisons, which are represented by the following alphabets. ‘a’—LF vs. HF; ‘b’—LF vs. HFr/HF; ‘c’—HF vs. HFr/HF.

Targeted metabolomics of organic acids and amino acids in the liver was conducted under fed and overnight fasted conditions. Under fed conditions, several of the metabolic intermediates were higher in the HFr/HF livers relative to their HF counterparts, but only pyruvate and citrate achieved statistical significance at *p* ≤ 0.05 (Figure 4A).

However, following an overnight fast, the hepatic content of several of the metabolic intermediates tended to be lower in the HFr/HF mice compared to their HF counterparts, with lactate, aspartate, succinate, malate, and fumarate significantly different between these groups at *p* ≤ 0.05 (Figure 4B). While these changes in fed and fasted metabolomic profiles do not allow us to determine the directionality of changes in mitochondrial metabolism, they indeed suggested a differential adaptation or remodeling of metabolism between HF and HFr/HF livers.

### 2.6. Alteration in the Pool Sizes of Hepatic TCA Cycle Intermediates Following Incubation of Isolated Mitochondria in a Respiration Buffer

**Figure 5:**
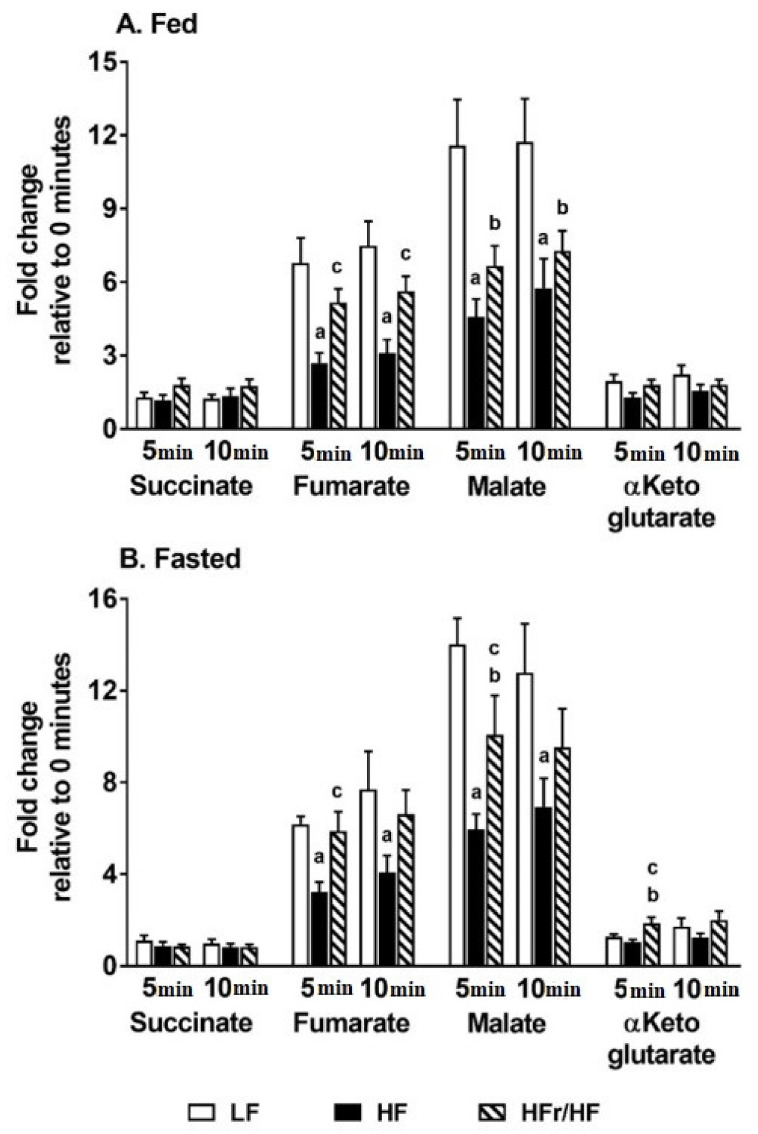
Changes in the pool sizes of hepatic TCA cycle intermediates with mitochondrial respiration. Profiles of organic acids in respiring liver mitochondria isolated from (**A**) fed and (**B**) overnight fasted livers, following 5 and 10 min of incubation. Data are presented as fold changes in the concentrations of metabolites at 5 and 10 min relative to their concentrations before incubation (0 min) of the mitochondria in a respiration buffer. Results (*n* = 7–11/group) were considered significant at *p* ≤ 0.05 following pairwise mean comparisons, which are represented by the following alphabets. ‘a’—LF vs. HF; ‘b’—LF vs. HFr/HF; ‘c’—HF vs. HFr/HF.

Stimulating respiration in isolated mitochondria over 10 min resulted in a steady increase in the organic acid intermediates, which serve as substrates of the TCA cycle. We utilized this premise to determine whether TCA cycle activity is different in mitochondria isolated from HF and HFr/HF livers. Under fed conditions, a general trend was evident where the pool sizes of several mitochondrial organic acids were higher in the HFr/HF group compared to the HF group, following 5 and 10 min of incubation (Figure 5A).

However, only the pool sizes of fumarate (at 5 and 10 min) achieved statistical significance at *p* ≤ 0.05 (Figure 5A). A similar trend of the TCA cycle intermediates being higher in the HFr/HF mitochondria following incubation was evident in the fasted mitochondrial incubations (Figure 5). In the fasted state, levels of fumarate, malate, and α-ketoglutarate were significantly higher (*p* ≤ 0.05) in the HFr/HF group vs. the HF group at 5 min of mitochondrial incubations (Figure 5B). Overall, these results suggest that the isolated mitochondria from the HFr/HF liver were more actively respiring compared to their HF counterparts, as illustrated by the greater pool sizes of the TCA cycle intermediates at 5 and 10 min of incubation.

### 2.7. Incorporation of ^13^C into Mitochondrial TCA Cycle Intermediates from [^13^C_3_]Pyruvate

**Figure 6:**
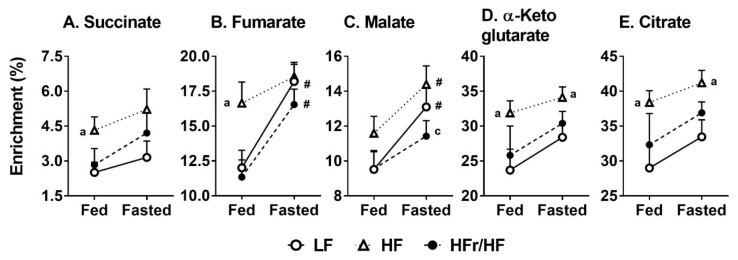
Enrichment of ^13^C in hepatic TCA cycle intermediates following the incubation of isolated mitochondria with [^13^C_3_]pyruvate. ^13^C enrichments in (**A**) succinate, (**B**) fumarate, (**C**) malate, (**D**) α-ketoglutarate, and (**E**) citrate from mitochondria isolated from livers of mice reared on LF, HF, and HC/HF diets. Results (*n* = 7–11/group) were considered significant at *p* ≤ 0.05 following pairwise mean comparisons, which are represented by the following alphabets. ‘a’—LF vs. HF; ‘c’—HF vs. HFr/HF. ‘#’ indicates significance at *p* ≤ 0.05 following a *t*-test between fed and fasted ^13^C enrichments.

To further substantiate our results from Figure 5, a second aliquot of isolated liver mitochondria from the three dietary groups was incubated with [^13^C_3_]pyruvate, and the incorporation of ^13^C was followed into the TCA cycle intermediates using GC-MS (Figure 6). Under both fed and fasted conditions, the incorporation of ^13^C into succinate, malate, α-ketoglutarate and citrate tended to be higher in the mitochondria isolated from the HF group. Furthermore, there was also a general trend where fasting increased the incorporation of ^13^C into several of the mitochondrial intermediates in comparison to the fed state. The fold change in ^13^C enrichment from feeding to fasting tended to be higher in the HFr/HF group, even though statistical significance (*p* ≤ 0.05) was observed only for fumarate and malate (Figure 6B,C). Taken together with the greater pool sizes of the TCA cycle intermediates at 5 and 10 min of incubation in the HFr/HF group (Figure 5), these results suggest that mitochondrial TCA cycle activity could be induced to a greater degree by the HFr/HF macronutrient environment.

### 2.8. Rates of Hepatic Mitochondrial Respiration Were Higher in Overnight Fasted HFr/HF Mice

**Figure 7:**
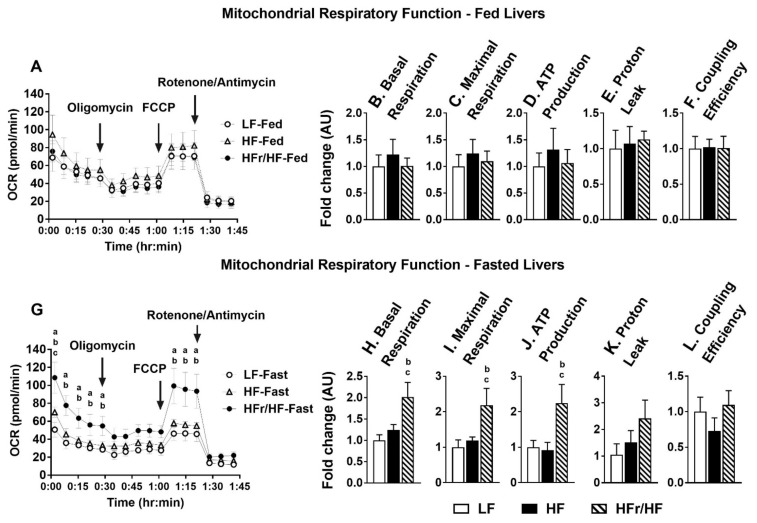
Hepatic mitochondrial respiratory function during fed and overnight fasting conditions. (**A**) Oxygen consumption rates (OCR) during basal conditions and following sequential treatment of mitochondria isolated from the livers of ‘fed’ mice, with oligomycin, FCCP, and rotenone/antimycin. Indices of ‘fed’ mitochondrial function determined from OCR measurements including (**B**) Basal respiration, (**C**) Maximal respiration, (**D**) ATP production, (**E**) Proton leak, and (**F**) Coupling efficiency. (**G**) OCR following the sequential treatment of mitochondria isolated from the livers of ‘fasted’ mice, with oligomycin, FCCP and rotenone/antimycin. Indices of ‘fasted’ mitochondrial function determined from OCR measurements including (**H**) Basal respiration, (**I**) Maximal respiration, (**J**) ATP production, (**K**) Proton leak, and (**L**) Coupling efficiency. Results (*n* = 5–6/group) were considered significant at *p* ≤ 0.05 following pairwise mean comparisons, which are represented by the following letters. ‘a’—LF vs. HF; ‘b’—LF vs. HFr/HF; ‘c’—HF vs. HFr/HF.

Schematic representation of oxygen consumption rates (OCR) under basal, oligomycin, FCCP, and rotenone/antimycin under fed and overnight fasted states are presented in Figure 7A,G. No significant differences in the indices of mitochondrial respiration were apparent between the three groups under fed conditions (Figure 7B–F). However, under fasting conditions, basal respiration, maximal respiration, and ATP production were higher in the HFr/HF mice (*p* ≤ 0.05) compared to their HF counterparts (Figure 7H–J). No statistical significance was observed between the groups following an overnight fast in proton leak and coupling efficiency (Figure 7K–L). Overall, these results point to higher fasting-induced mitochondrial respiratory activity in the livers of the HFr/HF mice.

### 2.9. Expression of Genes Involved in Hepatic Lipid Oxidation and Mitochondrial Reactive Oxygen Species (ROS) Generation Were Higher in HFr/HF Mice

**Figure 8:**
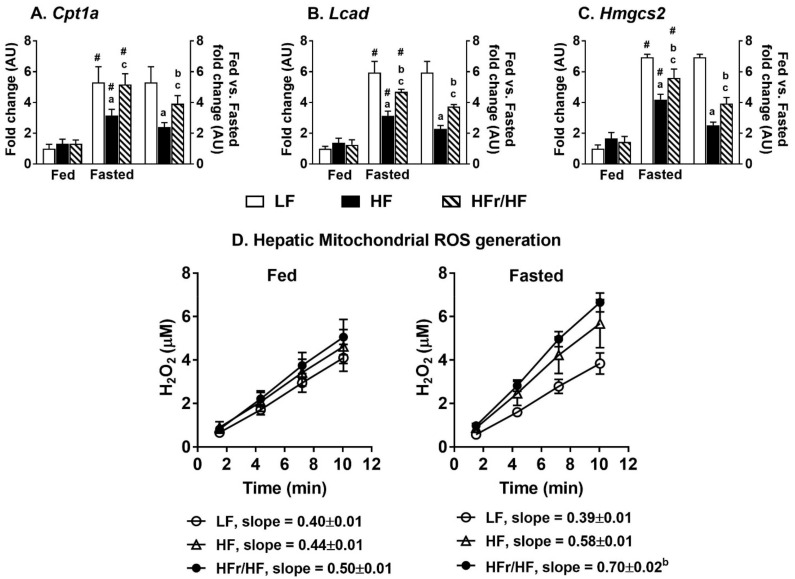
Expression of genes involved in hepatic lipid oxidation and measurements of mitochondrial ROS production. Expression of (**A**) Cpt1a, carnitine palmitoyltransferase 1a (**B**) Lcad, Long-chain acyl-CoA dehydrogenase, and (**C**) Hmgcs2, 3-hydroxy-3-methylglutaryl-Coenzyme A synthase 2 in the liver of fed and overnight fasted mice following LF, HF, and HC/HF dietary treatments. (**D**) Generation of ROS by the mitochondria isolated from livers of LF, HF, and HC/HF mice following feeding and overnight fasting conditions. Results (*n* = 7–11/group) were considered significant at *p* ≤ 0.05 following pairwise mean comparisons, which are represented by the following letters. ‘a’—LF vs. HF; ‘b’—LF vs. HFr/HF; ‘c’—HF vs. HFr/HF. ‘#’ indicates significance at *p* ≤ 0.05 following a *t*-test between fed and fasted gene expression.

Hepatic gene expression remained similar between groups under fed conditions (Figure 8A–C). Overnight fasting resulted in the induction of genes involved in hepatic lipid oxidation (*p* ≤ 0.05) in all the three groups of mice (Figure 8A–C). However, the fold induction of genes from feeding to fasting was significantly higher in the HFr/HF group (*p* ≤ 0.05), compared to their HF counterparts (Figure 8A–C). Furthermore, mitochondrial ROS generation, while similar between groups under fed conditions, tended to be higher in the HFr/HF group following an overnight fast (Figure 8D). The hepatic gene expression profiles corroborate the higher mitochondrial activity in the mice reared on HFr/HF diets.

## 3. Discussion

The metabolic plasticity of the liver allows it to efficiently shift from carbohydrate oxidation and lipogenesis during fed conditions, to lipid oxidation, ketogenesis, and gluconeogenesis under fasting conditions. With the onset of insulin resistance, these metabolic networks undergo progressive remodeling, eventually culminating in the failure to adapt to nutrient and hormonal cues [3,4,5,13,18,29]. With the progression of insulin resistance and NAFLD, metabolic dysfunction could manifest as the sustained induction of certain metabolic networks including hepatic lipogenesis, gluconeogenesis, and TCA cycle [3,13,18]. Depending on the severity of NAFLD, there can be a concurrent blunted fed-to-fasted induction of ketogenesis, impaired ATP synthesis, and/or reduced mitochondrial OXPHOS activity [18,30,31,32,33]. Hepatic mitochondria are at the center of this metabolic remodeling during NAFLD, due to their critical roles in integrating oxidative networks (β-oxidation, TCA cycle, ketogenesis, and OXPHOS) with anabolic networks of gluconeogenesis and lipogenesis. Dietary macronutrient composition has a major role in regulating the activity of these metabolic networks and can differentially impact mitochondrial metabolism and oxidative stress responses [34]. For example, insulin resistance and NAFLD induced by a carbohydrate-rich dietary environment (e.g., high-fructose diet) invariably activates lipogenesis [15,17,21], whereas high-fat diet-induced NAFLD could result in the suppression of the lipogenic machinery [19,20]. Furthermore, diets rich in fat, refined carbohydrates (e.g., fructose, sucrose) or both macronutrients can induce NAFLD [6,17,18,26,27]. At the same time, there is general consensus that the sustained activity of components of hepatic oxidative metabolism (e.g., TCA cycle) is a regular feature of diet-induced NAFLD [3,4,5,18]. These observations call into consideration whether the metabolic dysfunction fueled by two different macronutrient environments, which are known to promote NAFLD (HFr/HF vs. HF) could differentially impact the remodeling of hepatic mitochondrial oxidative function.

Our custom HFr/HF diet, containing 35% calories from fructose and 25% of the calories from lipids, induced significantly higher rates of de novo lipogenesis in the liver compared to the HF (60% fat calories) diet. It is also important to highlight that the rates of de novo lipogenesis in the liver of the HF mice were significantly lower than their LF (10% fat calories) counterparts. These results highlight the robust suppression of hepatic de novo lipogenesis by the high-fat dietary environment, which is commonly used in rodent models to induce obesity and NAFLD [35,36]. Thus, the high-fat diet-induced pathophysiology of NAFLD in rodent models occurs without any increase in de novo lipogenesis, which is a major component in the etiology of human NAFLD [13,37]. Interestingly, rates of de novo lipogenesis remained similar between LF and HFr/HF groups. This could be explained by the higher intake of lipid calories by the HFr/HF mice and these higher lipids suppressing lipogenesis in the liver. In spite of the contrasting rates in the de novo lipogenic flux, both high-fat and high-fructose dietary environments are known to aggravate insulin resistance and mitochondrial dysfunction during NAFLD [4,15,17,18]. Mice reared on the HF and HFr/HF diets showed characteristic symptoms of diet-induced insulin resistance onset, albeit only to a mild degree. We had deliberately set the time line on the custom diets to 10 weeks, to recreate earlier stages of hepatic insulin resistance and NAFLD, to investigate the initial remodeling of mitochondrial metabolism [18,25]. In turn, this allowed us to investigate the differential impact of these dietary environments on the early remodeling of hepatic mitochondrial oxidative function during NAFLD onset.

We rationalized that normal feeding-to-fasting metabolic transition will induce hepatic mitochondrial function [38], amplifying any oxidative defects in insulin-resistant livers. As expected, pyruvate and lactate levels in plasma were higher under fed conditions), which is a reflection of high glycolytic flux. Under fasting conditions, plasma ketones were significantly higher, which is a reflection of high rates of lipid oxidation. Interestingly, the reduction in serum pyruvate and lactate levels and the increase in serum ketone levels in response to fasting were blunted in the HF mice compared to the mice on the HFr/HF diets. We interpreted this blunted response as evidence for a less efficient switch from carbohydrate utilization to free fatty acid utilization during the fed-to-fasted transition in the HF mice. This interpretation was also supported by the significantly lower fed-to-fasted fold induction of genes involved in hepatic lipid catabolism in HF mice compared to the HFr/HF mice. Furthermore, OXPHOS complex III, IV, and V proteins were also higher in the liver of the HFr/HF mice, suggesting that the hepatic mitochondria in these mice were better primed to upregulate their oxidative machinery during fasting. The targeted metabolomics of hepatic mitochondrial intermediates also illustrated significant differences between the HFr/HF vs. HF groups under both fed and fasting states, suggesting differences in mitochondrial function between these two groups. Taken together, the high-fructose/high-fat environment had a differential impact on hepatic mitochondrial function compared to those observed on a high-fat rich environment.

The remodeling of mitochondrial oxidative function is a dynamic process, especially considering the fact that the oxidative machinery comprises of multiple biochemical networks including β-oxidation, TCA cycle, ketogenesis, OXPHOS, and ATP synthesis. These biochemical networks do not remodel in the same direction or at the same rate during the progression of NAFLD [11,39,40]. During the initial stages of hepatic insulin resistance and NAFLD onset, it is believed that in response to a macronutrient overload, mitochondrial oxidative networks overcompensate, leading to an initial induction in nutrient flux through certain oxidative networks, including ketogenesis and the TCA cycle [5,18]. Furthermore, with prolonged severity of the disease, certain mitochondrial networks are blunted in their activity, while certain others stay sustained. For example, we and others have demonstrated an induction in rates of ketogenesis, TCA cycle flux, and lipid oxidation during early stages of hepatic insulin resistance [3,5,18,24]. With the progressive severity of insulin resistance and NAFLD, ketogenic flux is reduced, while the TCA cycle activity remains higher [18]. A similar adaptation could occur with OXPHOS where mitochondrial respiratory function is higher during milder stages of insulin resistance and lower/impaired during more severe stages of the disease [5,32]. There is also evidence of reduced capacity for ATP production during NAFLD [33]. It is important to understand the remodeling of mitochondrial oxidative fluxes during NAFLD under various macronutrient environments, as the chronic activity of these oxidative networks will fuel and sustain higher rates of ROS generation and inflammation [9,16]. Following this rationale, we tracked the remodeling of mitochondrial oxidative function in liver from HFr/HF and HF dietary environments. Contrary to our hypothesis, our results suggest that the activity of oxidative networks is higher in the liver mitochondria from the HFr/HF mice compared to their HF counterparts. This conclusion was corroborated by several of our results. (1) The fold induction of ketones with fasting was higher in the HFr/HF mice. (2) The rate of production of TCA cycle intermediates following mitochondrial respiration was higher in the HFr/HF livers. (3) The expression profiles of lipid oxidation genes in the liver were higher in the HFr/HF mice. (4) The OXPHOS activity was higher in the HFr/HF livers. Consistent with the rationale that higher mitochondrial oxidative function will parallel higher ROS generation, mitochondria from the HFr/HF mice livers had higher rates of ROS production. Taken together, we conclude that a dietary environment that is concurrently high in fructose and lipid calories recapitulates the macronutrient composition of Western diets. The metabolic milieu in the HFr/HF livers promote higher mitochondrial oxidative activity, together with higher rates of lipogenesis, during the initial stages of hepatic insulin resistance.

In summary, our results suggest that the variations in dietary macronutrient environments can differentially influence the rate of mitochondrial oxidative remodeling and lipogenesis. The impact of these results is in the fact that these differences in mitochondrial remodeling can influence the rate of progression of NAFLD and the severity of hepatic insulin resistance, which are modulated by various dietary environments. The dietary treatments (HFr/HF vs. HF), which were applied for 10 weeks in this study, only resulted in mild hepatic insulin resistance; thus, they are reflective of the early stages of NAFLD onset, when the initial remodeling of mitochondrial oxidative metabolism is active. Thus, the observed induction of mitochondrial oxidative function associated with the high-fructose/high-fat environment could be an early compensatory response to the macronutrient overload. How the prolonged induction of mitochondrial oxidative function, fueled by the HFr/HF diet, impacts the progressive severity of NAFLD needs to be further explored.

## 4. Materials and Methods

### 4.1. Animals and Diets

This study was approved by the Institutional Animal Care and Use Committee at the University of Maryland, College Park, MD. Male C57BL/6NJ mice were procured from Jackson Laboratory (Bar Harbor, ME, USA) at 4–6 weeks of age. Once mice were around 8 weeks old, the standard chow was replaced with either a low-fat control diet (LF; 10% kcal fat, 20% kcal protein, and 70% kcal carbohydrate; D12450J), high-fat diet (HF; 60% kcal fat, 20% kcal protein, and 20% kcal carbohydrate; D12492), or high fructose/high-fat (HFr/HF; 25% kcal fat, 20% kcal protein, and 34.9% kcal fructose; D19013101). The majority of lipid calories in these custom diets were derived from animal fat (lard), the free fatty acid composition of which is well known [41]. All the diets were customized by Research Diets (New Brunswick, NJ, USA; Table S1), and the mice were placed on these custom diets for 10 weeks. These dietary formulations reflect a range of macronutrient compositions, with the HF and the HFr/HF dietary environments projected to contribute to the onset and severity of NAFLD. The mice were further randomly divided into fed and overnight fasted (15–16 h) groups, prior to the metabolic profiling studies detailed below. The sample size utilized for the specific set of experiments is highlighted under their respective figures.

### 4.2. Hepatic Mitochondrial Isolation

Mice were anesthetized using isoflurane and blood collected from the posterior venacava, which was then spun at 2500 g for 10 min to separate the serum, which was stored at minus 80 °C for later analysis. Fresh liver was collected in ice-cold phosphate-buffered saline (PBS; 1X) and washed with ice-cold PBS. Around 0.6–1.0 g fresh liver was minced in 3 mL of MSHE buffer (70 mM sucrose, 210 mM mannitol, 5 mM HEPES, 1 mM EGTA, and 0.5% fatty acid-free bovine serum albumin (BSA); pH 7.2). The minced liver was transferred to a 30 mL Dounce homogenizer and homogenized with four strokes; the first having 10 turns, the second and third having 5 turns, and the fourth having 2 turns. The homogenate was transferred to an ice-cold 15 mL falcon tube. The Dounce homogenizer was rinsed with an additional 4 mL of MSHE, which was mixed with the homogenate. Then, the liver homogenate was centrifuged for 10 min at 4 °C at 800× *g*. The supernatant was collected in a fresh ice-cold 15 mL falcon tube after passing through a double-layered cheesecloth pre-soaked in ice-cold MSHE buffer. The supernatant was centrifuged at 8000× *g* for 10 min at 4 °C. The supernatant was discarded; then, the pellet was re-suspended in 3 mL of ice-cold MSHE and centrifuged at 8000× *g* for 10 min at 4 °C. The process is repeated with 2 mL of MSHE buffer and the remaining pellet, containing approximately 2.5 to 3.5 mg of mitochondrial protein re-suspended in 100 µL MSHE buffer without BSA. The protein concentration was measured using the Pierce protein assay kit (Thermo Fisher Scientific, Waltham, MA, USA).

### 4.3. Incubation of Isolated Mitochondria to Determine Changes in TCA Cycle Metabolism

Mitochondria (500 µg based on protein measurements) was incubated in 1 mL MAS-3 buffer (115 mM KCl, 10 mM KH_2_PO_4_, 2 mM MgCl_2_, 3 mM HEPES, 1 mM EGTA and 0.2% fat free BSA; pH 7.2) containing either (1) 5 mM glutamate, 2.5 mM malate, and 1 mM uniformly labeled [^13^C_3_] pyruvate or (2) 5 mM glutamate, 2.5 mM malate, and 1 mM unlabeled pyruvate. Mitochondrial protein aliquots from each liver were incubated for 0, 5, and 10 min at 37 °C. These time points were selected to stimulate mitochondrial respiration and the subsequent changes in mitochondrial TCA cycle intermediates, as a reflection of mitochondrial activity. Mitochondrial respiratory activity was stopped by placing samples on ice for 1 min before centrifugation at 3000× *g* for 5 min at 4 °C. The supernatant was collected in a separate Eppendorf tube, and the pellet was washed with 250 µL of ice-cold PBS. The pellet was centrifuged again at 3000× *g* for 2 min at 4 °C, and the supernatant was discarded. The pellet was covered with 100 µL cold methanol and stored for the determination of ^13^C enrichments in the TCA cycle intermediates by GC-MS. The second aliquot of mitochondria incubated with unlabeled pyruvate was prepared similarly to determine the concentrations of TCA cycle intermediates by GC-MS.

### 4.4. Metabolite Profiling by GC-MS

The TCA cycle intermediates in the liver tissue (≈15 mg) and isolated mitochondrial protein (500 µg) were extracted with 750 µL of chloroform: methanol (2:1) and 250 µL of water. The aqueous phase was dried under nitrogen, and the TCA cycle intermediates were converted to their oximes with 20 µL of 2% methoxamine hydrochloride in pyridine (*w*/*v*) by microwaving for 90 s, which is followed by conversion to their respective TBDMS (Tert-butyldimethylsilyl) derivatives [42]. Serum (25 µL) samples were processed following deproteinization with 750 µL of cold acetonitrile and the conversion of metabolites to their oxime with 20 µL of 2% methoxamine hydrochloride in pyridine, followed by TBDMS derivatization. For the determination of metabolite concentrations, liver tissue, mitochondria, and serum samples were spiked with a known amount of stable isotope labeled internal standards of the respective metabolites. Metabolites were separated on a HP-5MS UI column (30 m × 0.25 mm × 0.25 μm; Agilent, Santa Clara, CA, USA) and fragmented under electrical ionization. Fragments of interest were detected using single ion monitoring (SIM) on a GC-MS (5973N-Mass Selective Detector, 6890-Series GC, Agilent, Santa Clara, CA, USA) [42].

For determination of serum glucose levels, a stable isotope labeled internal standard of glucose was spiked into 25 µL of serum before deproteinization by 750 µL of cold acetonitrile. The extracted glucose was dried and converted to its di-O-isopropylidene acetate derivative and separated on a fused silica capillary column (HP-5; 30 m × 0.25 mm i.d., 0.25 µm; Agilent, Santa Clara, CA, USA) with helium as the carrier gas, followed by selective ion monitoring on MS (5973N Mass Selective Detector coupled to a 6890 Series GC System, Agilent, Santa Clara, CA, USA) under electrical ionization conditions [43].

Liver glycogen pellets were isolated as previously described [43]. Briefly, 25 mg of liver was deproteinized with 1 mL of ice-cold sulfosalicylic acid and glycogen extracted by the addition of ice-cold ethanol to the supernatant (2:1), and centrifugation at 10,000× *g* for 15 min. The isolated glycogen pellet was washed two times with ice-cold ethanol and the free glucose units were liberated by incubation (1 h at 55 °C) in 250 μL of buffer (0.3 M acetic acid and 2 M acetate; 1:1; pH 4.5) containing 0.5 mg of amyloglucosidase (31.2 units/mg of solid). The incubation mixture was lyophilized to dryness, and the di-O isopropylidene acetate derivative of glucose was formed before GC-MS analysis as described above. Liver glycogen was estimated as free glucose units liberated from the amyloglucosidase activity relative to a known amount of stable isotope labeled glucose internal standard spiked into the sample.

### 4.5. Biochemical Assays

Serum NEFA concentrations were determined using the NEFA-HR2 analytical kit (WAKO diagnostics, Richmond, VA, USA. Serum insulin was determined using the Ultra-sensitive mouse insulin ELISA kit (Crystal Chem, Downers Grove, IL, USA). Liver triglycerides from approximately 20 mg of tissue were Folch extracted in 750 µL of chloroform:methanol (2:1). The extracted triglycerides were dried down before determining the triglyceride content using the triglyceride determination kit (Sigma Aldrich, St. Louis, MO, USA) with triolein as the standard.

### 4.6. Determination of ROS Production by Isolated Mitochondria

The rate of H_2_O_2_ production in mitochondria was measured using the oxidation of the fluorogenic indicator amplex red (10-acetyl-3,7-dihydroxyphenoxazine) in the presence of horseradish peroxidase (HRP). Mitochondria (64 µg) were incubated at 37 °C with HRP (0.2 U/mL) and amplex red reagent (100 µM) prepared in MAS-3 buffer with 5 mM glutamate and 2.5 mM malate. Fluorescence was recorded using the Cytation 5 spectrophotometer (BioTek Instruments, Inc. Winooski, VT, USA) with 530 nm excitation and 590 nm emission wavelengths. Standard curves were constructed by adding known amounts of H_2_O_2_ to the assay medium in the presence of the reactants.

### 4.7. Respiration by Isolated Liver Mitochondria

Changes in the respiratory function of the isolated hepatic mitochondria were determined using the Seahorse XF Cell Mito Stress Test Kit (Agilent Technologies, Inc, Santa Clara, CA, USA). Modulators of mitochondrial oxidative phosphorylation, namely Oligomycin (5 μM) F0-F1ATPase inhibitor, carbonylcyanide *p*-(trifluoromethoxy), phenylhydrazone (FCCP, 4.95 μM), antimycin A, and rotenone (2.53 μM) were utilized for this test. Isolated mitochondria (10 µg/well) were added to the chilled test cell plate containing the media (1:1, MAS-3 with 20 mM palmitoyl carnitine and 2.5 mM malate: MSHE without BSA) and centrifuged at 2000× *g* for 20 min at 4 °C. Then, the plate was incubated at 37 °C for 10 min before starting the Cell Mito Stress Test. The OCR were measured using Seahorse xFP analyzer (Agilent, Santa Clara, CA, USA) as follows: no equilibration, 5 basal readings, 5 readings with oligomycin, 3 readings with FCCP, and 3 readings with rotenone and antimycin. Indices of mitochondrial respiratory function were determined from the OCR values utilizing the calculations provided by the manufacturer.

### 4.8. Gene Expression Analysis

Total RNA was extracted from 20 mg snap-frozen liver tissue using 500 µL Trizol reagent (Invitrogen, Carlsbad, CA, USA) and mRNA mini-prep kit (Bio-Rad Laboratories Inc., Hercules, CA, USA). The cDNA was reverse transcribed from 1 μg mRNA using cDNA synthesis kit (Bio-Rad, Hercules, CA, USA). Quantitative real-time PCR was performed by amplifying 25 ng of cDNA, 150 nM of forward and reverse primers (Table S2), and 5 μL of SYBR green PCR master mix (Invitrogen, Carlsbad, CA, USA) in a 10 μL reaction with cyclophilin (Ppib) as the house keeping gene. Samples were run in triplicate on a Bio Rad CFX Real Time system C1000 Touch Thermal Cycler (Bio-rad, Hercules, CA, USA). The mRNA abundance was determined relative to the housekeeping gene, cyclophilin A.

### 4.9. Western Blot Analysis

Liver (around 20 mg) and mitochondrial homogenates were lysed with protease and phosphatase inhibitors. Protein content was measured using a Pierce BCA protein assay kit (Thermo Fischer Scientific. Waltham, MA, USA). Liver and mitochondrial OXPHOS proteins were separated using Bolt 8% Bis-tris Plus gels (Invitrogen, Carlsbad, CA, USA), transferred to a nitrocellulose membrane, and blocked with 5% milk in tris-buffered saline with Tween 20. Then, the membranes were probed with the primary antibodies of interest including Akt, pAkt^S473^, COX IV, and GAPDH (Cell Signaling technology Inc., Danvers, MA, USA). A Total OXPHOS Rodent WB Antibody Cocktail (Abcam plc. Cambridge, MA, USA) was used to profile mitochondrial complex proteins involved in oxidative phosphorylation. Images were collected using a Gel Doc XR+ system (Bio-rad, Hercules, CA, USA).

### 4.10. Statistical Analysis

Based on our previous experience, we expected the standard deviation for our measurements to range between 20 and 30%. With the statistical power (1-β) set at 80% and an alpha error of 5%, we calculated a minimum sample size requirement of five mice per group. All the data reported are presented as means ± standard error of means (SEM). All results (except the mitochondrial respiration data in Figure 7) were analyzed using one-way ANOVA, and multiple pair-wise mean comparisons were performed by controlling the false discovery rate using the two stage step-up method of Benjamini, Krieger, and Yekutieli. Mitochondrial respiration data were analyzed using repeated measures and two-way ANOVA with Fisher’s least significant difference test. Means ± SEM were considered significantly different at *p* ≤ 0.05. All statistical analyses were conducted and the graphs were plotted utilizing Prism 7 (Graph Pad Software Inc., San Diego, CA, USA).

## Figures and Tables

**Table 1:** Characteristics of mice reared on LF, HF, and HFr/HF diets for 10 weeks.

	Feeding Status	LF	HF	HFr/HF
Body Weight (g)	Fed	29.0 ± 0.6	42.9 ± 1.1 ^a^	33.4 ± 1.1 ^bc^
	Fasted	26.2 ± 0.8	35.1 ± 1.4 ^a^	31.7 ± 1.2 ^b^
Food Intake (kcal/day)		9.97 ± 0.14	12.95 ± 0.19 ^a^	12.30 ± 0.18 ^bc^
Liver Weight (g)	Fed	1.43 ± 0.05	1.84 ± 0.18 ^a^	1.74 ± 0.08 ^b^
	Fasted	1.10 ± 0.05	1.15 ± 0.05	1.21 ± 0.06
Plasma Glucose (mM)	Fed	7.38 ± 0.24	7.70 ± 0.29	7.31 ± 0.24
	Fasted	4.76 ± 0.36	5.99 ± 0.33 ^a^	5.20 ± 0.36
Liver Glycogen (mg/g liver)	Fed	52.7 ± 4.6	50.5 ± 4.5	64.3 ± 3.4 ^c^
	Fasted	5.0 ± 1.9	8.6 ± 1.7	2.4 ± 0.6^c^
Plasma Insulin (ng/mL)	Fed	1.24 ± 0.40	3.83 ± 1.13	2.92 ± 1.20
	Fasted	0.42 ± 0.20	0.59 ± 0.18	0.78 ± 0.25
Plasma NEFA (mM)	Fed	0.34 ± 0.05	0.52 ± 0.03 ^a^	0.37 ± 0.04 ^c^
	Fasted	0.92 ± 0.04	0.78 ± 0.04 ^a^	0.78 ± 0.05
Liver TG (mg/g liver)	Fed	23.1 ± 2.0	31.1 ± 2.7 ^a^	23.5 ± 1.7 ^c^
	Fasted	41.2 ± 3.8	35.3 ± 2.2	34.9 ± 2.3
Total Liver TG (mg)	Fed	33.4 ± 3.6	60.3 ± 12.3	40.9 ± 3.7
	Fasted	46.2 ± 5.7	40.6 ± 3.0	43.5 ± 4.5
Inguinal Adipose Tissue Weight (g)	Fed	0.92 ± 0.14	2.74 ± 0.25 ^a^	1.56 ± 0.15 ^bc^
	Fasted	0.78 ± 0.22	2.00 ± 0.42 ^a^	1.58 ± 0.20 ^b^
HOMA-IR		2.45 ± 1.32	4.07 ± 1.41	5.29 ± 2.06

All the values (*n* = 5 for inguinal adipose tissue and *n* = 9–11 mice for all other measurements) are expressed as means ± SEM. Significance at *p* ≤ 0.05 following pairwise mean comparisons are represented by the alphabets. ‘a’—LF vs. HF; ‘b’—LF vs. HC/HF; ‘c’—HF vs. HC/HF. TG, triglycerides; NEFA, non-esterified fatty acids.

## Data Availability

All data are contained within the article or supplementary material.

## Supplementary Materials

The following are available online at https://www.mdpi.com/article/10.3390/metabo11050272/s1, Figure S1: Gene expression profiles of canonical markers of inflammation and Pgc1a in livers isolated from mice under ‘Fed’ state, Table S1: Composition of the rodent diets, Table S2: Primer sequences used for qPCR measurements.

## References

[1] Rinella M.E. (2015). Nonalcoholic Fatty Liver Disease. JAMA.

[2] Mantovani A., Byrne C.D., Bonora E., Targher G. (2018). Nonalcoholic Fatty Liver Disease and Risk of Incident Type 2 Diabetes: A Meta-analysis. Diabetes Care.

[3] Sunny N.E., Parks E.J., Browning J.D., Burgess S.C. (2011). Excessive Hepatic Mitochondrial TCA Cycle and Gluconeogenesis in Humans with Nonalcoholic Fatty Liver Disease. Cell Metab..

[4] Patterson R.E., Kalavalapalli S., Williams C.M., Nautiyal M., Mathew J.T., Martinez J., Reinhard M.K., McDougall D.J., Rocca J.R., Yost R.A. (2016). Lipotoxicity in steatohepatitis occurs despite an increase in tricarboxylic acid cycle activity. Am. J. Physiol. Metab..

[5] Koliaki C., Szendroedi J., Kaul K., Jelenik T., Nowotny P., Jankowiak F., Herder C., Carstensen M., Krausch M., Knoefel W.T. (2015). Adaptation of Hepatic Mitochondrial Function in Humans with Non-Alcoholic Fatty Liver Is Lost in Steatohepatitis. Cell Metab..

[6] García-Berumen C.I., Ortiz-Avila O., Vargas-Vargas M.A., Del Rosario-Tamayo B.A., Guajardo-López C., Saavedra-Molina A., Rodríguez-Orozco A.R., Cortés-Rojo C. (2019). The severity of rat liver injury by fructose and high fat depends on the degree of respiratory dysfunction and oxidative stress induced in mitochondria. Lipids Health Dis..

[7] Bronson F.H. (1987). Susceptibility of the fat reserves of mice to natural challenges. J. Comp. Physiol. B.

[8] Bronson F.H., Heideman P.D., Kerbeshian M.C. (1991). Lability of fat stores in peripubertal wild house mice. J. Comp. Physiol. B.

[9] Chance B., Sies H., Boveris A. (1979). Hydroperoxide metabolism in mammalian organs. Physiol. Rev..

[10] Selen E.S., Choi J., Wolfgang M.J. (2021). Discordant hepatic fatty acid oxidation and triglyceride hydrolysis leads to liver disease. JCI Insight.

[11] Shannon C.E., Ragavan M., Palavicini J.P., Fourcaudot M., Bakewell T.M., Valdez I.A., Ayala I., Jin E.S., Madesh M., Han X. (2021). Insulin resistance is mechanistically linked to hepatic mitochondrial remodeling in non-alcoholic fatty liver disease. Mol. Metab..

[12] Donnelly K.L., Smith C.I., Schwarzenberg S.J., Jessurun J., Boldt M.D., Parks E.J. (2005). Sources of fatty acids stored in liver and secreted via lipoproteins in patients with nonalcoholic fatty liver disease. J. Clin. Investig..

[13] Lambert J.E., Ramos–Roman M.A., Browning J.D., Parks E.J. (2014). Increased De Novo Lipogenesis Is a Distinct Characteristic of Individuals with Nonalcoholic Fatty Liver Disease. Gastroenterology.

[14] Ter Horst K.W., Vatner D.F., Zhang D., Cline G.W., Ackermans M.T., Nederveen A.J., Verheij J., Demirkiran A., Van Wagensveld B.A., Dallinga-Thie G.M. (2021). Hepatic Insulin Resistance Is Not Pathway Selective in Humans With Nonalcoholic Fatty Liver Disease. Diabetes Care.

[15] Todoric J., Di Caro G., Reibe S., Henstridge D.C., Green C.R., Vrbanac A., Ceteci F., Conche C., McNulty R., Shalapour S. (2020). Fructose stimulated de novo lipogenesis is promoted by inflammation. Nat. Metab..

[16] Satapati S., Kucejova B., Duarte J.A., Fletcher J.A., Reynolds L., Sunny N.E., He T., Nair L.A., Livingston K.A., Fu X. (2016). Mitochondrial metabolism mediates oxidative stress and inflammation in fatty liver. J. Clin. Investig..

[17] Softic S., Gupta M.K., Wang G.-X., Fujisaka S., O’Neill B.T., Rao T.N., Willoughby J., Harbison C., Fitzgerald K., Ilkayeva O. (2017). Divergent effects of glucose and fructose on hepatic lipogenesis and insulin signaling. J. Clin. Investig..

[18] Satapati S., Sunny N.E., Kucejova B., Fu X., He T.T., Méndez-Lucas A., Shelton J.M., Perales J.C., Browning J.D., Burgess S.C. (2012). Elevated TCA cycle function in the pathology of diet-induced hepatic insulin resistance and fatty liver. J. Lipid Res..

[19] Duarte J.A., Carvalho F., Pearson M., Horton J.D., Browning J.D., Jones J.G., Burgess S.C. (2014). A high-fat diet suppresses de novo lipogenesis and desaturation but not elongation and triglyceride synthesis in mice. J. Lipid Res..

[20] Green C.J., Pramfalk C., A Charlton C., Gunn P.J., Cornfield T., Pavlides M., Karpe F., Hodson L. (2020). Hepatic de novo lipogenesis is suppressed and fat oxidation is increased by omega-3 fatty acids at the expense of glucose metabolism. BMJ Open Diabetes Res. Care.

[21] Stanhope K.L., Schwarz J.M., Keim N.L., Griffen S.C., Bremer A.A., Graham J.L., Hatcher B., Cox C.L., Dyachenko A., Zhang W. (2009). Consuming fructose-sweetened, not glucose-sweetened, beverages increases visceral adiposity and lipids and decreases insulin sensitivity in overweight/obese humans. J. Clin. Investig..

[22] Rodríguez-Calvo R., Barroso E., Serrano L., Coll T., Sánchez R.M., Merlos M., Palomer X., Laguna J.C., Vázquez-Carrera M. (2008). Atorvastatin prevents carbohydrate response element binding protein activation in the fructose-fed rat by activating protein kinase A. Hepatology.

[23] Softic S., Cohen D.E., Kahn C.R. (2016). Role of Dietary Fructose and Hepatic De Novo Lipogenesis in Fatty Liver Disease. Dig. Dis. Sci..

[24] Iozzo P., Bucci M., Roivainen A., Någren K., Järvisalo M.J., Kiss J., Guiducci L., Fielding B., Naum A.G., Borra R. (2010). Fatty Acid Metabolism in the Liver, Measured by Positron Emission Tomography, Is Increased in Obese Individuals. Gastroenterology.

[25] Sunny N.E., Satapati S., Fu X., He T., Mehdibeigi R., Spring-Robinson C., Duarte J., Potthoff M.J., Browning J.D., Burgess S.C. (2010). Progressive adaptation of hepatic ketogenesis in mice fed a high-fat diet. Am. J. Physiol. Metab..

[26] Ishimoto T., Lanaspa M.A., Rivard C.J., Roncal-Jimenez C.A., Orlicky D.J., Cicerchi C., Mcmahan R.H., Abdelmalek M.F., Rosen H.R., Jackman M.R. (2013). High-fat and high-sucrose (western) diet induces steatohepatitis that is dependent on fructokinase. Hepatology.

[27] Fonseca C.S.M., Basford J.E., Kuhel D.G., Konaniah E.S., Cash J.G., Lima V.L.M., Hui D.Y. (2020). Distinct Influence of Hypercaloric Diets Predominant with Fat or Fat and Sucrose on Adipose Tissue and Liver Inflammation in Mice. Molecules.

[28] Cordain L., Eaton S.B., Sebastian A., Mann N., Lindeberg S., A Watkins B., O’Keefe J.H., Brand-Miller J. (2005). Origins and evolution of the Western diet: Health implications for the 21st century. Am. J. Clin. Nutr..

[29] Boland M.L., Oldham S., Boland B.B., Will S., Lapointe J.-M., Guionaud S., Rhodes C.J., Trevaskis J.L. (2018). Nonalcoholic steatohepatitis severity is defined by a failure in compensatory antioxidant capacity in the setting of mitochondrial dysfunction. World J. Gastroenterol..

[30] Nair S., Chacko V.P., Arnold C., Diehl A.M. (2003). Hepatic ATP reserve and efficiency of replenishing: Comparison between obese and nonobese normal individuals. Am. J. Gastroenterol..

[31] Cortez–Pinto H., Lin H.Z., Yang S.Q., da Costa‡ S.O., Diehl A.M. (1999). Lipids up-regulate uncoupling protein 2 expression in rat hepatocytes. Gastroenterology.

[32] Perez-Carreras M., Del Hoyo P., Martin M.A., Rubio J.C., Martin A., Castellano G., Colina F., Arenas J., Solis-Herruzo J.A. (2003). Defective hepatic mitochondrial respiratory chain in patients with nonalcoholic steatohepatitis. Hepatology.

[33] Einer C., Hohenester S., Wimmer R., Wottke L., Artmann R., Schulz S., Gosmann C., Simmons A., Leitzinger C., Eberhagen C. (2018). Mitochondrial adaptation in steatotic mice. Mitochondrion.

[34] Simoes I.C.M., Karkucinska-Wieckowska A., Janikiewicz J., Szymanska S., Pronicki M., Dobrzyn P., Dabrowski M., Dobrzyn A., Oliveira P.J., Zischka H. (2020). Western Diet Causes Obesity-Induced Nonalcoholic Fatty Liver Disease Development by Differentially Compromising the Autophagic Response. Antioxidants.

[35] Aydos L.R., Amaral L.A.D., De Souza R.S., Jacobowski A.C., Dos Santos E.F., Macedo M.L.R. (2019). Nonalcoholic Fatty Liver Disease Induced by High-Fat Diet in C57bl/6 Models. Nutrients.

[36] Kakimoto P.A., Kowaltowski A.J. (2016). Effects of high fat diets on rodent liver bioenergetics and oxidative imbalance. Redox Biol..

[37] Smith G.I., Shankaran M., Yoshino M., Schweitzer G.G., Chondronikola M., Beals J.W., Okunade A.L., Patterson B.W., Nyangau E., Field T. (2020). Insulin resistance drives hepatic de novo lipogenesis in nonalcoholic fatty liver disease. J. Clin. Investig..

[38] Rui L. (2014). Energy Metabolism in the Liver. Compr. Physiol..

[39] Léveillé M., Estall J.L. (2019). Mitochondrial Dysfunction in the Transition from NASH to HCC. Metabolites.

[40] Sunny N.E., Bril F., Cusi K. (2017). Mitochondrial Adaptation in Nonalcoholic Fatty Liver Disease: Novel Mechanisms and Treatment Strategies. Trends Endocrinol. Metab..

[41] Liu M., Lampi A.-M., Ertbjerg P. (2018). Unsaturated fat fraction from lard increases the oxidative stability of minced pork. Meat Sci..

[42] Muyyarikkandy M.S., McLeod M., Maguire M., Mahar R., Kattapuram N., Zhang C., Surugihalli C., Muralidaran V., Vavilikolanu K., Mathews C.E. (2020). Branched chain amino acids and carbohydrate restriction exacerbate ketogenesis and hepatic mitochondrial oxidative dysfunction during NAFLD. FASEB J..

[43] Surugihalli C., Porter T.E., Chan A., Farley L.S., Maguire M., Zhang C., Kattapuram N., Muyyarikkandy M.S., Liu H.-C., Sunny N.E. (2019). Hepatic Mitochondrial Oxidative Metabolism and Lipogenesis Synergistically Adapt to Mediate Healthy Embryonic-to-Neonatal Transition in Chicken. Sci. Rep..

