# Supplementary material for: Dietary Macronutrient Composition Differentially Modulates the Remodeling of Mitochondrial Oxidative Metabolism during NAFLD
Source: Metabolites. 2021 Apr 26;11(5):272. doi: 10.3390/metabo11050272 (PMC8147090; doi:10.3390/metabo11050272)
Supplement: Supplementary file 1 [file metabolites-11-00272-s001.zip › metabolites-1149932-supplementary.pdf]

Table S1. Composition of the rodent diets.

	Low Fat (LF)		High Fat (HF)		High Fructose/ High Fat (HFr/HF)	
	**g%**	***kcal%***	**g%**	***kcal%***	**g%**	***kcal%***
Protein	19	20	26	20	21	20
Carbohydrate	67	70	26	20	57	55
Fat	4	10	35	60	12	25
Total		100		100		100
kcal/g	3.8		5.2		4.2	
**Ingredient**	**g**	***kcal***	**g**	***kcal***	**g**	***kcal***
Casein	200	800	200	800	200	800
L-Cystine	3	12	3	12	3	12
Corn Starch	506	2025	0	0	0	0
Maltodextrin 10	125	500	125	500	125	500
Sucrose	68.8	275	68.8	275	68.8	275
Fructose	0	0	0	0	353.8	1415
Cellulose, BW200	50	0	50	0	50	0
Soybean Oil	25	225	25	225	25	225
Lard	20	180	245	2205	87.7	789
Mineral Mix, S10026	10	0	10	0	10	0
DiCalcium Phosphate	13	0	13	0	13	0
Calcium Carbonate	5.5	0	5.5	0	5.5	0
Potassium Citrate, 1 H_2O	16.5	0	16.5	0	16.5	0
Vitamin Mix, V10001	10	40	10	40	10	40
Choline Bitartrate	2	0	2	0	2	0
Total	1055.0	4057	773.8	4057	970.3	4057

Table S2. Primer sequences used for qPCR measurements.

Gene	Forward	Reverse
Acc1	GGACAGACTGATCGCAGAGAAAG	TGGAGAGCCCCACACACA
Fasn	GCTGCGGAAACTTCAGGAAAT	AGAGACGTGTCACTCCTGGACTT
Scd1	GCGATACACTCTGGTGCTCA	CCCAGGGAAACCAGGATATT
Elovl6	TGCCATGTTCATCACCTTGT	TGCTGCATCCAGTTGAAGAC
Cpt1a	CAAAGATCAATCGGACCCTAGAC	CGCCACTCACGATGTTCTTC
Lcad	TCAATGGAAGCAAGGTGTTCA	GCCACGACGATCACGAGAT
Hmgcs2	GACTTCCTGTCATCCAGC	GGTGTAGGTTTCTTCCAGC
Tnfa	CTGAGGTCAATCTGCCCAAGTAC	CTTCACAGAGCAATGACTCCAAAG
Il6	TCGTGGAAATGAGAAAAGAGTTG	AGTGCATCATCGTTGTTCATACA
Nfkbia	GAGCAAATGGTGAAGGAG	TCTGGAAGTTGAGGAAGG
Pgc1a	AGACAAATGTGCTTCCAAAAAGAA	GAAGAGATAAAGTTGTTGGTTTGGC
Ppib	GGAGATGGCACAGGAGGAA	GCCCGTAGTGCTTCAGCTT

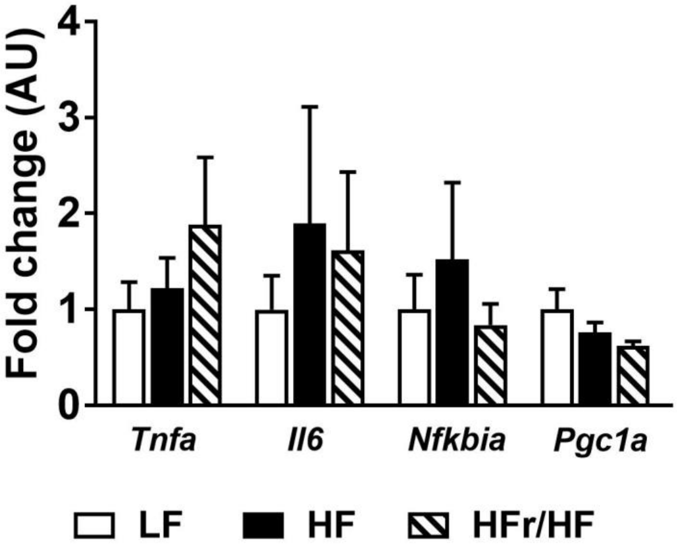

Figure S1. Gene expression profiles of canonical markers of inflammation and Pgc1a in livers isolated from mice under 'Fed' state. Results (n = 5/group) were considered significant at $p \leq 0.05$.
